# A policy analysis of the national phenylketonuria screening program in Iran

**DOI:** 10.1186/s12913-021-06116-w

**Published:** 2021-02-05

**Authors:** Alireza Heidari, Mohammad Arab, Behzad Damari

**Affiliations:** 1 Health Management and Social Development Research Center, Golestan University of Medical Sciences (GOUMS), Golha Alley, Gorgan, Iran; 2 Department of Management and Health Economic, School of Public Health, Tehran University of Medical Sciences (TUMS), Tehran, Iran; 3 Governance and Health Department, Neuroscience Institute, Tehran University of Medical Sciences (TUMS), Tehran, Iran

**Keywords:** Policy analysis, Phenylketonuria (PKU), Neonatal screening, Policy triangulation framework, National PKU screening (NaPS)

## Abstract

**Background:**

Phenylketonuria (PKU) screening is a public health measure taken to diagnose and treat the patients with PKU to prevent severe neurological disorders in them. The present study was aimed at analyzing the policies of the national PKU screening (NaPS) program in Iran.

**Methods:**

PKU screening program policies were analyzed in compliance with the policy triangle model. Document review and 38 semi-structured interviews were used for data collection. Document review data were analyzed using content analysis, and interview data were analyzed using framework analysis.

**Results:**

The national PKU screening (NaPS) program was a decision made at the genetics department of Ministry of Health and Medical Education (MOHME) in Iran. Many internal and external stakeholders were involved in it and valid evidence was used to formulate the policies. Despite some opposition and insufficient support, the program was implemented due to the continuous persistence of parents, interested executives, formulated valid content and a top-down approach. The main barriers included rapid substitution of managers, shortage of Phe-free milk, little awareness of patients’ families, social stigma, and inadequate co-operation of some hospital administrators.

**Conclusions:**

The policy triangle framework contributed to explaining the different components of the PKU screening program. A successful PKU screening program requires more stability of senior managers in MOHME, enough human resources and Phe-free milk, educating patients’ families, and commitment of hospitals administrators. Meanwhile, all the stakeholders need to be involved in the program effectively.

**Supplementary Information:**

The online version contains supplementary material available at 10.1186/s12913-021-06116-w.

## Background

Phenylketonuria (PKU) is the most common hereditary metabolic disorder in the world [1]. Patients with PKU show high levels of phenylalanine in the blood serum, and thus develop neurological disorders such as mental retardation, seizures, behavioral problems, and developmental delays [2–4]. The prevalence of the disease is reported to be 1 in 10,000 in whites and 1 to 6000–8000 in the Iranian population [5]. Other domestic studies have also reported a high incidence of the disease among people with mental retardation from 2.1 to 5% [6, 7]. If the disease is not diagnosed and treated within the first year of birth, the child’s IQ capacity may be reduced by 50%. Irreversible effects on the brain can be prevented by timely detection and control of phenylalanine levels in the blood [8].

Neonatal screening is a population-based public health screening program implemented for early detection [9]. PKU screening is a prerequisite for early implementation of the Phe restricted diet, which is essential to prevent severe neurological disorders in patients with PKU [10]. Nowadays, most developed countries carry out PKU screening, and any newborn with PKU is immediately exposed to a Phe-restricted diet to lower plasma Phe concentrations. This combination of early diagnosis and initiation of treatment results in normal IQ for most PKU patients [11].

The PKU screening program in Iran launched in 2006. In this program blood samples were taken from all infants on day 3 to 5 after birth for colorimetric screening. And people with phenylalanine levels of 4 mg/dl or higher were referred to be confirmed by HPLC test. Then regular follow-up is performed for people who have phenylalanine levels equal to or greater than 4 mg/dl, and if the phenylalanine levels are 7 mg/dl or more, the diet begins with restricted phenylalanine. Dietary supplements of Iron, zinc, selenium, carnitine, vitamins and essential fatty acids are prescribed for all children up to 2 years of age [12].

Policy analysis includes plans and actions aimed at achieving health care goals by defining a vision for the future, setting goals and objectives, setting priorities, and identifying the roles of different groups. Health policy analysis helps to understand the success or failure in implemented policies so that they can be useful for planning future policies. Health policy analysis helps policymakers to improve their successful implementation of future policies and provide opportunities to produce policy documents [13]. Policy analysis can help to understand the complexity of the policy process as well as its nature and provide policy-related evidence [14]. Policy analysis helps to understand why policymakers pay attention to certain issues in the health system and disregard other issues, also to identify stakeholders who agree or disagree with the policies and the reasons why they agree or disagree with it, as well as to identify undesirable consequences of policies implementation and future problems in implementing policies and achieving their goals [15, 16]. Since, studying the role of evidence in policymaking helps to better understand the contribution of research to policy formation and to identify the factors that influence it [17] and no sufficient works have been conducted on policy analysis of the phenylketonuria screening, the present study is aimed at analyzing the policies of the national phenylketonuria screening (NaPS) program in Iran.

## Methods

### Study design

**Fig. 1:**
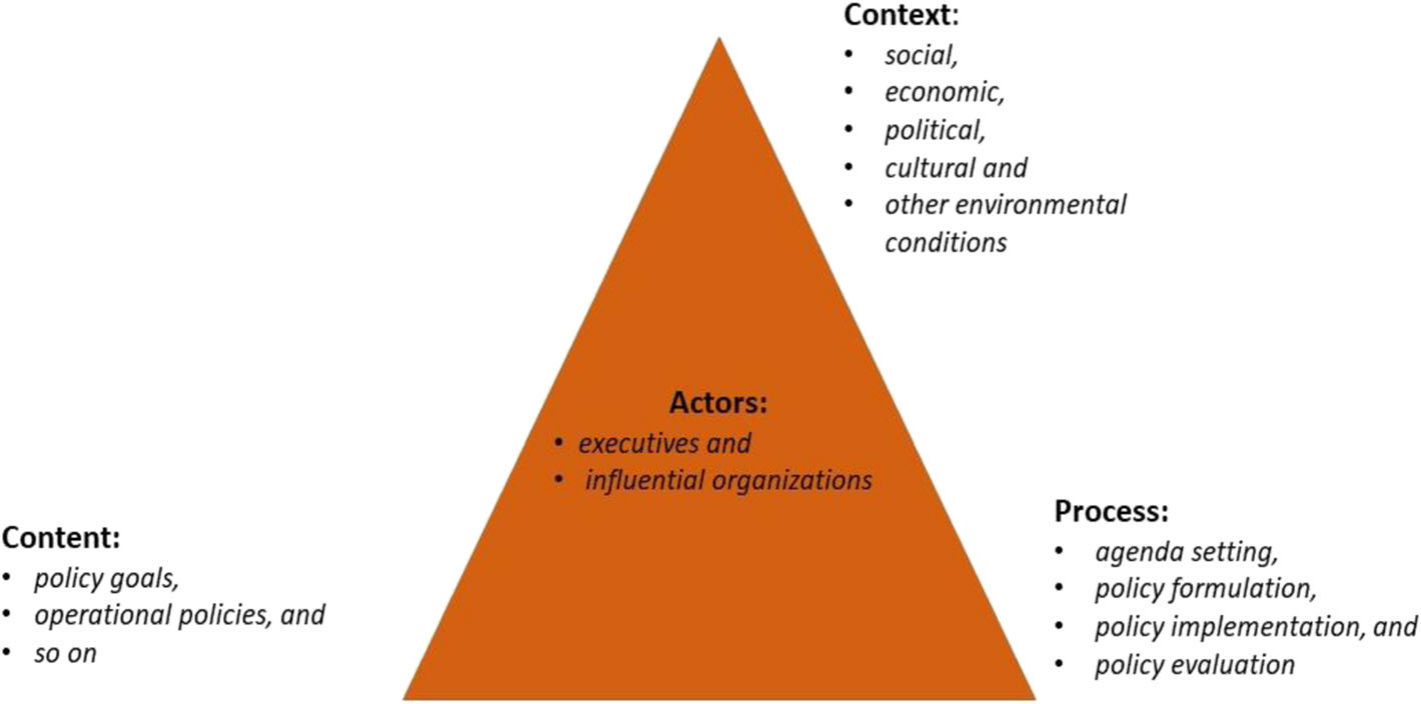
Basic health policy analysis model based on Walt and Gilson triangle framework

This qualitative study was conducted from May 2015 to January 2016 using the Walt and Gilson triangle Kingdon’s multiple streams models. Kingdon model is an instrument for understanding the policies agenda-setting. Based on this model, the interaction of three streams problem, policy and politics will result in a policy window and policy entrepreneurs can use the opportunities for policy development [18]. The triangle model covers four general areas: content, context, actors, and the decision-making process (Fig. 1). The *content* includes policy goals, operational policies, and so on. The word “*actors*” refers to executives and influential organizations. The *context* refers to social, economic, political, cultural and other environmental conditions. The *process* consists of four parts: agenda setting, policy formulation, policy implementation, and policy evaluation [19].

### Study population and selection of participants

Study participants included 38 individuals from different sectors related to the study in Iran:

- the Health Commission of the Islamic Consultative Assembly,
- Ministry of Health and Medical Education (MOHME) (deputies of health and treatment, food and drug administration),
- the Iranians Health Insurance organization,
- the policymaking council,
- faculty members in universities of medical sciences,
- children’s specialized hospitals,
- Pasteur Institute,
- Welfare Organization, and
- PKU Patients Support Association.

Target groups were selected based on purposive sampling. Their selection criteria included knowledge and experience in phenylketonuria screening programs, active participation in phenylketonuria screening programs, and interest in research participation.

### Data collection

Semi-structured interviews were conducted to collect data using the interview guideline. Credibility, transferability, dependability, and conformability were used such as criteria of rigor and trustworthiness of this study [20–22]. Interviews were conducted face-to-face at a designated time and place. The interviews lasted between 30 and 65 min. At the beginning of each interview, the participants were provided with a summary of the research topic and the method of using the data. The written consent was obtained to conduct the interview. Note taking was also used during the recording of the interviews. The audio files were transcribed at the earliest opportunity.

### Data analysis

Framework Analysis was used to analyze the data, which included five basic steps: Familiarization, Identifying a Thematic Framework, Indexing, Charting and Mapping and Interpretation. In addition, Document Analysis was used to validate the findings of the interviews and to benefit from the evidence available in the policy process. Documents were collected purposefully by referring to the organizations involved in NaPS program and reviewing relevant internet sites. The content analysis method was used for data analysis of documents.

## Results

**Fig. 2:**
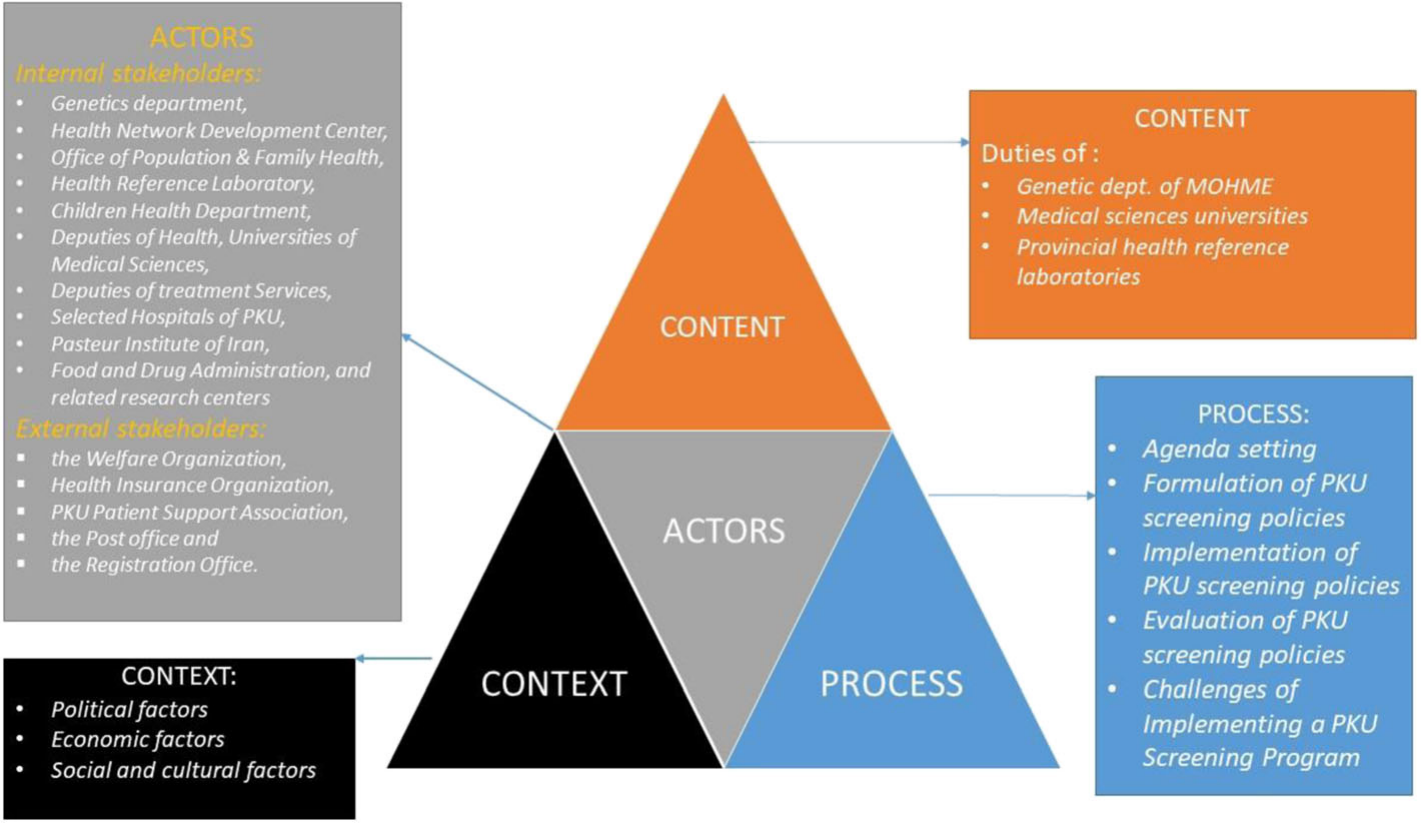
The policy framework of National Phenylketonuria Screening (NaPS) Program in Iran based on *Walt & Gilson* Triangle Framework

Findings were reported based on four categories of policy triangle framework (context, content, process, and actors). These categories are explained as follows (Fig. 2):

### Context

Content included three factors: political, economic, and social and cultural factors.

#### Political factors

Political commitment and stability of top-level healthcare administrators of MOHME will contribute to the continuity of the approach of fair access to health services for all. According to Article 89 of Iran’s Fourth Development Plan (1963–1972), the MOHME was required to design a system of minimum standardization of health services based on the levels of services in order to ensure fair access to health services. In this regard, the NaPS program with specific level was launched at the lowest levels of the network, was fairly implemented in cities and villages. But the long-term plan for the future has not been defined. Because of the political changes in government, the minister, his deputies and other senior managers of the MOHME change relatively fast.

*"The system should not change with the change of managers. It must be stable. After the management changes, specialized staff also change. Then the problem emerges "*(P.19).

#### Economic factors

Economic sanctions will exert the most pressure on the needy and less privileged /under-privileged and susceptible patients- those who cannot afford the diagnostic and therapeutic costs of PKU. The economic sanctions restricted the currency exit from country and the imports of drugs and Phe-free milk and even publication of scientific articles related to the PKU program in foreign journals. Because most families could not afford the diagnostic and therapeutic costs of the disease, the cost of screening and Phe-free milk was subsidized by government.

*“Iran sanctions face us with limited drug access and low access to technology”* (P.17).

“*Sanctions have had a major impact on the import of Phe-free milk for patients*”(P.4).

*“We had trouble publishing the article on this project, so one of the journals responding to our article replied: we cannot publish your article because you are under sanctions”* (P.24).

#### Social and cultural factors

Adequate public education on PKU- e.g. mental retardation, prevention, and follow up- and knowledge of healthcare providers on the PKU-associated social stigma will help decrease the impacts of the disorder and achieve the goals of the program. People welcomed the diagnosis program as it dealt with concerns about the likelihood of mental retardation and the possibility of preventing the disease, but some families do not regularly follow up on treatment regimens. Parenting skills of patients’ parents and self-care of patients are poor. Patients and their families have little knowledge of the disease, and they are not provided with adequate education. The tendency for family marriages in Iran leads to an increased risk of the disease. The social stigma associated with disease has made it difficult to advance the program.

*“With age, withdrawal from treatment increases and disease control becomes more difficult. Many patients develop seizures because they have not followed the diet and phenylalanine levels in their blood have risen.”* (P.28).

*“Disease-related social stigma makes it difficult to care and follow up. The parents of one of the patients moved and they would not be followed up as they do not provide their new address. A person who has a child with PKU doesn’t even tell his sister and brother that his child has PKU”* (P.36).

### Policy content

To help NaPS program to be effective, different specialized and administrative departments at country, provincial and national levels and universities need to fulfil their duties, responsibilities, and follow regulations in this regard. The purpose of NaPS program was to reduce physical disability, mental retardation and family damage. PKU screening is not just a test, but it is a system designed to identify, treat, and fully care for patients. Therefore, the program was organized at county, provincial, and national levels.

The genetics department of the MOHME and medical sciences universities were determined as responsible for implementing the program in national and provincial levels. In health part, health houses, urban and rural health centers, district health centers and disease prevention and control unit in the deputy of health of the medical sciences universities were considered to provide health services. In treatment part, blood sampling centers, provincial health reference laboratory and selected PKU hospitals were considered to provide diagnostic and therapeutic services.

Chief among the duties of genetics department are:

- drafting the national program guidelines,
- developing the monitoring checklists,
- forming the national technical committees,
- notifying all universities in the country of the program,
- collecting the annual reports from medical sciences universities,
- evaluating implemented programs,
- following up the necessary facilities,
- engaging relevant organizations and departments and making coordination with them,
- following up laws and approvals,
- developing the educational packages,
- anticipating funding for program implementation and reporting to high level officials.

Chief among the duties of medical sciences universities are:

▪ planning and discussing the program at the genetics committee,

▪ organizing meetings of the PKU academic committee,

▪ monitoring implementation of approvals,

▪ monitoring performance, and

▪ periodically reporting to health and treatment deputies.

The duties of the provincial health reference laboratory are:

❖ receiving and registering all screening samples,

❖ performing all screening tests,

❖ reporting positive screening tests (initial suspected) and

❖ requiring re-testing (technical and medical) to the “disease prevention and control unit” in the province.

The number of patients in each province is relatively limited, so such a large system cannot be implemented in all cities. In this way, at least one children’s specialized hospital as PKU selected hospital in each province provides all required services to patients. In PKU selected hospitals, all required services are provided:

- ○ specialist physician periodic visit,
- ○ laboratory services for treatment control,
- ○ expert nutrition services,
- ○ pharmaceutical services (delivery of Phe-free milk and other nutrition items to patients),
- ○ clinical psychology and social care services, and
- ○ parents’ education.

The duties of the health centers and health houses include:

- immediate referral of newly-diagnosed patients to the selected hospitals,
- follow-up in absence of treatment, collection, registration and transmission of information,
- participation in educational programs, and
- educating pregnant mothers.

In order to implement the program, guidelines of laboratory, care, clinical, nutritionist, genetic diagnosis and prevention, non-classic sampling guidelines and supplementary regulations of clinical psychologists were developed.

### Policy process

The results of policy process of PKU program consists of five sections: 1) Agenda setting, 2) Formulation of PKU screening policies, 3) Implementation of PKU screening policies, 4) Evaluation of PKU screening policies, and 5) Challenges of implementing a PKU screening program.

#### Agenda setting

Three different streams are needed to set up an agenda for the policy process of PKU program:

1. Problem Stream: Identifying the problems behind the necessity of implementing a national PKU screening program;
2. Policy Stream: Support of insurance organizations and treatment department of MOHME; and

3. Political Stream: Adequate support of patients’ families and members of the Iranian Society of Pediatrics to implement NaPS program.

During 1997–2010, the outbreak ratio of PKU was calculated 1:8000 in three big provinces of Tehran, Fars, and Mazandaran in Iran [8]. Following the implementation of the Iranian NaPS in 2013, the incidence rate of this disease was decreased to 1:4166 [23]. If the disease is not diagnosed in a timely manner and the treatment process is not initiated, physical, mental and intellectual disabilities will occur for the patient and these problems are irreversible. The medical costs and problems of keeping a disabled child in the family are also other problems (*First Stream*).

“*There was a problem. Every year a number of patients were added to PKU patients. They suffered from severe physical and mental disabilities, reduced IQs and complications such as seizures and increased financial burden for the families and health system. On the other hand, the disease could be diagnosed early and be cared for*” (P.1).

The above-mentioned problems have led health policymakers to explore ways to solve these problems. Therefore, with the successful implementation of the hypothyroid screening program and the use of infrastructure of this program for phenylketonuria screening, as well as executive and scientific efforts, PKU screening program was developed (*Second stream*).

*“We had already experienced congenital hypothyroidism screening. Sampling time, sampling method and sending to the lab are the same as phenylketonuria screening”* (P.14).

Although, there were some problem and policy streams, factors such as lack of prioritization of the program by the MOHME top executives, the limited capacity of the health system, the inability to increase health personnel in accordance with national administrative regulations and laboratory equipment problems, caused the policy window to remain closed and program implementation stopped.

Despite the opposition of the Network Development Office with regard to integrating the program, insufficient support of insurance organizations and deputy of treatment of MOHME, the NaPS program was implemented due to the continuous persistence of patients’ families and members of the Iranian Society of Pediatrics to initiate screening and therapeutic interventions and formulated content, and talented and interested executives who convinced the policymakers (*Third stream*).

“*We, patients’ families, corresponded with the Vice-Chancellor of Health Minister. We met him. We talked to him about our problems. He was very upset about our situation. He instructed the relevant organizations to serve all PKU patients”* (P.22).

“*One of the reasons for the success of this program was the presence of a capable, interested, and persistent responsible person*” (P.16).

Problems associated with the disease (*first stage*), successful implementation of the hypothyroid screening program and ability to run the program based on the created infrastructure at national level (*second stage*), exerting pressure on the patients’ families, members of the Scientific Committee on Children and following up by executives (*third stage*) concurrently led to agreement of health policy makers with the program, and policy window was opened.

#### Formulation of PKU screening policies

To formulate the policies of PKU screening, various departments need to implement different administrative, scientific and executive measures to achieve this goal. Executives sought to involve all stakeholders prior to launching the program. The scientific proposal for the implementation of the screening program was formulated by two faculty members. A services package to be provided to patients with hereditary metabolic diseases was developed by faculty members working with various medical sciences universities in Iran. The Genetics Department used the Thalassemia Genetics Program as the infrastructure and model of the PKU program and utilized thalassemia genetic consultants. The standards were extracted from the review of scientific references, valid guidelines and evidences from leading countries and then localized in the national committee. In order to formulate policies, numerous meetings were held with the participation of academics and stakeholders to engage them to advance the program. WHO observers attended some of these meetings. The program was presented in the Provincial Health and Food Security Council by health administrators. In order to ensure the feasibility of implementing the program, diagnostic and therapeutic facilities were checked out by different groups of genetic experts, physicians, and health reference laboratory experts.

#### Implementation of PKU screening policies

Different approaches should be used to implement the PKU screening policies. The approach for implementing PKU screening policies was a top-down approach. The pilot program was conducted in 2007 at Universities of Medical Sciences in Shiraz, Mazandaran and 3 Universities of Medical Sciences in Tehran, i.e. Shahid Beheshti, Tehran and Iran University of Medical Sciences. The screening program was nationwide following the implementation of the pilot one in early 2013. Organizing the laboratory tests, transferring the specimens and confirming the tests were done by the Health Reference Laboratory. In the capital of each province, a children’s specialized hospital was organized as PKU’s selected hospital. A treatment team consisting of a pediatrician, a nutritionist, a psychologist, a social worker, and a secretary was formed in these hospitals. Patients’ dietary milk was distributed by the selected hospital pharmacy. At the beginning of the program, a liaison was selected from among the patients’ parents, but after a while, this role was assigned to the hospital staff. The follow-up of the newborn baby was monitored by health center experts. Detailed statistics of treatment and absence of treatment from the selected hospital were provided to the MOHME. Malignant and non-classic diagnostic tests were conducted at the Pasteur Institute of Iran.

#### Evaluation of PKU screening policies

Provincial experts’ performance was evaluated by the Genetic Department of MOHME. In this way, periodic monitoring and completing checklists and providing statistics and documentation was done.

#### Challenges of implementing a PKU screening program

Different sections of a health care system should actively cooperate to fulfil the successful implementation of PKU screening program. A national screening decision was made in 2011, but was delayed due to laboratory equipment problems in medical sciences universities in 2012. Monitoring and follow-up of PKU patients have lower priority than other programs in some selected hospitals. Selected hospitals suffered from a shortage of milk powder. It was not appropriate to record hospital and non-hospital information. Qualitative evaluation of selected hospitals was not performed well due to inadequate co-operation of some hospital administrators and the lack of a supervisory system for providing PKU services in hospitals.

“*From the year 2007 to 2011, when we wanted to start a national screening program, during these four years, the most important problems were laboratory diagnostic problems*” (P.3).

*“One of the problems is that hospital information is not recorded very well*” (P.2).

### Actors

Internal stakeholders involved in the NaPS program include:

- Genetics department,
- Health Network Development Center,
- Office of Population & Family Health,
- Health Reference Laboratory,
- Children Health Department,
- Deputies of Health Universities of Medical Sciences,
- Deputies of treatment Services,
- Selected Hospitals of PKU,
- Pasteur Institute of Iran,
- Food and Drug Administration,
- and related research centers

External stakeholders involved were:

- the Welfare Organization,
- Health Insurance Organization,
- PKU Patient Support Association,
- the Post office and
- the Registration Office.

## Discussion

The purpose of the Phenylketonuria screening program in Iran was to reduce physical disability and mental retardation and medical and nonmedical costs. Given the relatively high prevalence of PKU [23] compared to that of other countries [24–28], high rates of familial marriages in Iran [29, 30] and mental retardation in patients with PKU [31, 32] highlighted the role of early diagnosis of disease and the need for timely medical interventions. Establishing a specific mechanism for the care and control of PKU patients was also the main demand of the patients’ families, but despite the problems related to the disease and its consequences, due to the limited capacity of the health system, the inability to increase the number of human resources in accordance with national administrative regulations and lack of laboratory equipment, the policymakers and senior managers at the MOHME were not convinced to integrate the program into the health system and to implement the program at the national level. Eventually, after pressure from the demand and follow-up by patients’ parents and members of the Pediatric Medical Association, policy makers and senior executives were persuaded to implement the program. The results of Barojo study in 2007 also showed that policymakers’ willingness and pressure of families with affected children led to a particular disease being selected for the neonatal screening program in Latin American countries without any appropriate basis or the use of a national standard [33]. In this regard, in the United States, legal pressure from parents and the legislature was the main driving force behind the widespread screening [34].

Based on the results of the present study, to implement the program nationwide, provincial laboratories were equipped. The surveillance system for the affected patients was organized in health networks. PKU selected hospitals were designated in the capital of each province and a treatment team consisting of a pediatrician, a nutritionist, a psychologist, a social worker and a secretary was formed in these hospitals. There are some differences between Iranian structure of human resources for PKU screening and that of the other countries. New Zealand, which launched the Metabolic Disorders Screening Program since 2005, used a consulting group including pediatricians, patients’ families and other medical teams before implementing the program [35]. The results of the Hanley study in Canada in 2005 showed that health care workers for the phenylketonuria screening program were physicians, nutritionists, nurses, social workers, biochemists, genetic counselors, and psychologists [36]. Midwives, neonatal unit staff, health visitors and health team members, nursing specialist consultants, pediatric nurses in the pediatric nursing team, child health team, public health staff, general practitioners and pediatricians are involved in the implementation of the UK Neonatal Screening Program [37]. Benefitting from nurses and midwives in the care team of patients with PKU in Canada and the United Kingdom was a distinction between their human resources structure and the one practiced in Iran.

Based on the results, a “top-down” approach for policy implementation was adopted. Ham and Hill have considered two approaches for policy implementation; the main difference between these approaches is the degree of actors’ involvement in the policy cycle [38]. In the bottom-up approach, field actors can influence performance through negotiation and engagement, and in the “top-down” approach, bargaining and interaction between different levels of the policy cycle are low [39]. The “top-down” approach, i.e. the approach used in policy implementation, focuses on a small group of high-level policymakers at the MOHME, and policies are seen as high-level orders of the high-level authorities that the low-level authorities are forced to implement. Although periodic visits and reporting occur at different levels of the health care system, these interactions are not sufficient and may reduce the long-term participation of actors at lower levels. Inadequate co-operation of hospital administrators seems to be one of the consequences of applying this approach.

The results of the study indicated the impact of political changes in the country and consequently structural and managerial changes in the health system which have an adverse impact on the implementation of NaPS policies and programs. This result is in line with the results of the studies by Romain et al. in 2015 in Tunisia [40]. Managers and policies changes that occur after political changes may cause confusion and lead to halting the programs. Therefore, development of a long-term plan and the commitment of all managers to it will help to sustain the plan.

Another political factor was the impact of international sanctions on imports of dietary milk and medicine. Studies by Masoumi et al. in 2015 [41], Georgia et al. in 2014 [42] and Shahabi et al. in 2015 [43] have also addressed the impact of sanctions on health. The sanctions have had a negative impact on the country’s treatment system, which includes the provision of drugs and medical equipment. In many cases, it has impeded the entry of certain drugs and has also caused slow entry of drugs and equipment, the introduction of counterfeit and inappropriate drugs, and increased prices for other drugs [44].

One of the important cultural factors was the family’s lack of awareness of PKU. This result is consistent with the results of a study by Hong et al. in Southern Taiwan in 2005 [45] and Barojo study [32], but it was inconsistent with the results of Campbell and Ross’s study in the US in 2003 [46]. Parents’ ignorance of the disease can be due to their poor health literacy. Since the first step towards behavior change about a subject is to obtain awareness about it [47], therefore, parents knowledge should be increased based on the content of scientific guidelines to lead to effective care.

### Strengths and limitation of the study

This study had some strength. To use a qualitative approach, internal and external stakeholders participated in the study with different backgrounds and explained their own professional experience individually. Two well-known conceptual framework (policy analysis triangle and Kingdon’s multiple streams models) used were able to collect relevant data including process, content and context and actors to interpret development policies.

Nonetheless, this study suffers from some limitations. The study, rather than being longitudinal was a cross-sectional study. Since this study was the first research to analyze the PKU screening policies, other restrictions of the study was limited access to documents.

## Conclusion

The present study was conducted to analyze the policies of PKU Screening in Iran. The analysis helped to identify the different components of policymaking cycle and effective factors on formulation, implementation, and evaluation of policies. A successful PKU Screening program requires more stability of senior managers in the MOHME, enough human resources and Phe-free milk, educating patients’ families, and commitment of hospitals administrators and also, all of the stakeholders should be involved in the program effectively.

## Supplementary Information

**Additional file 1.**

## Data Availability

In order to preserve the anonymity of participants transcripts are not available. In this study, document review and 38 semi-structured interviews were used for data collection. Therefore, datasets are not available for the personal nature of the information. Data may be available upon justified request from the corresponding author with restrictions and following the ethical approval.

## Abbreviations

HPLC: High performance liquid chromatography
IQ: Intelligence Quotient
MOHME: Ministry of Health and Medical Education
NaPS: National Phenylketonuria Screening
PKU: Phenylketonuria
