# Supplementary material for: A policy analysis of the national phenylketonuria screening program in Iran
Source: BMC Health Serv Res. 2021 Feb 5;21:120. doi: 10.1186/s12913-021-06116-w (PMC7863318; doi:10.1186/s12913-021-06116-w)
Supplement: Supplementary file 1 — Additional file 1. [file 12913_2021_6116_MOESM1_ESM.docx]

.

**Interview Guide**

**Question 1**: Explain the situation of phenylketonuria (PKU) screening in Iran.

Exploration Question: Can you explain more about the epidemiological situation of the disease, the importance and necessity of screening program, the reasons for the program's priority, the main and specific goals of the program, the status of diagnostic and therapeutic facilities, the social acceptance of the program, the coverage of the program?

**Question 2**: Explain the policies and programs of PKU screening in Iran.

Exploration Question: Can you explain more about the structure, organization, guidelines, rules and duties of national, provincial, city levels?

**Question 3**: Which organizations and units are involved in the policies related to PKU in Iran? Explain.

Exploration question: Can you explain interaction of these organizations and departments with screening policies and programs, the role and position, the executive power, the level of communication of these organizations in policies formulation and implementation.

**Question 4:** How to formulate and implement policies and programs of PKU screening in Iran? Explain.

Exploration Question: can you explain more about how to develop and implement PKU screening policies and programs at the diagnostic, therapeutic, and health care levels.

**Question 5:** Explain the reasons for the success or failure of policies and programs of PKU screening in Iran.

Exploration Question: Can you explain more about the strengths, weaknesses, barriers and facilitators of PKU screening programs?

**Question 6**: Explain the effective economic, social, cultural and political factors that affect the success or failure of policies and programs of PKU screening in Iran.

Exploration Question: can you explain more about political and executive factors, economic and financial factors, social and cultural factors that affect the implementation of PKU screening policies.

**Question 7:** In the development of policies and programs of PKU screening in Iran, what global policies and programs have been studied? Explain.

Exploration Question: Can you explain more how much reliable protocols and scientific guidelines of reputable organizations and successful countries have been used and on PKU screening to design policies?
